# Exosome-based cell therapy for diabetic foot ulcers: Present and prospect

**DOI:** 10.1016/j.heliyon.2024.e39251

**Published:** 2024-10-11

**Authors:** Zhou Yang, Mengling Yang, Shunli Rui, Wei Hao, Xiaohua Wu, Lian Guo, David G. Armstrong, Cheng Yang, Wuquan Deng

**Affiliations:** a Department of Endocrinology and Metabolism, School of Medicine, Chongqing University Central Hospital, Chongqing Emergency Medical Center, Chongqing, 400014, China; b Department of Endocrinology, School of Medicine, Chongqing University Three Gorges Central Hospital, Chongqing, 404000, China; c Department of Surgery, Keck School of Medicine of University of Southern California, Los Angeles, CA, 90033, USA

**Keywords:** Diabetic foot ulcers, Wound healing, Exosomes, Membrane vesicles, Regenerative medicine

## Abstract

Diabetic foot ulcers (DFUs) represent a serious complication of diabetes with high incidence, requiring intensive treatment, prolonged hospitalization, and high costs. It poses a severe threat to the patient's life, resulting in substantial burdens on patient and healthcare system. However, the therapy of DFUs remains challenging. Therefore, exploring cell-free therapies for DFUs is both critical and urgent. Exosomes, as crucial mediators of intercellular communication, have been demonstrated potentially effective in anti-inflammation, angiogenesis, cell proliferation and migration, and collagen deposition. These functions have been proven beneficial in all stages of diabetic wound healing. This review aims to summarize the role and mechanisms of exosomes from diverse cellular sources in diabetic wound healing research. In addition, we elaborate on the challenges for clinical application, discuss the advantages of membrane vesicles as exosome mimics in wound healing, and present the therapeutic potential of exosomes and their mimetic vesicles for future clinical applications.

## 1 Introduction

Diabetic foot ulcers (DFUs), a serious complication of diabetes mellitus, are challenging to heal due to the susceptibility to infection, and a higher rate of recurrence, disability and mortality [1]. There are about 18.6 million diabetic foot ulcers sufferers worldwide annually, approximately 20 % of these individuals eventually require minor or major limb amputation, thus presenting a significant worldwide health problem [2]. Compared with non-diabetic skin wounds, DFUs exhibit impaired endothelial cell metabolism due to persistent hyperglycemia, blockage of angiogenesis, increased tissue inflammatory factors and reactive oxygen species (ROS), and difficulty in polarizing anti-inflammatory macrophage M2 [3,4]. Enhanced matrix metalloproteinase (MMP) protease activity leads to extracellular matrix (EMC) degradation, impaired function of keratinocytes and fibroblasts, and inhibited re-epithelialization. Previous studies also indicated that DFU wounds had increased amount of neutrophil extracellular traps (NETs), leading to worsened wound injury and infection [5]. Traditional treatments of DFUs include glycemic control, anti-infection, vascular dilation, anti-platelet, lower limb revascularization, surgical debridement, and wound management. The updated International Working Group on the Diabetic Foot (IWGDF) 2023 guideline for DFUs recommends the use of sucrose-octasulfate dressings, placental derivatives, autologous leukocytes, platelet and fibrin patches, negative pressure wound therapy, and hyperbaric oxygen to improve local hypoxia as adjuncts to promote healing of diabetic foot ulcers [6]. However, the persistently high morbidity, disability, and rising costs suggest that it is urgent to explore new treatment strategies.

In recent years, our team and others have been focusing on developing emerging strategies for diabetic wound healing, particularly involving exosomes derived from cells [7]. These exosomes carry genetic material, metabolic factors, lipids, and cell surface proteins inherited from cellular source [8]. They function in intercellular communication [9] and play a crucial role in the process of diabetic wounds healing [10,11]. Almost all cell types secrete nanometer-sized vesicles called exosomes, which measure between 30 nm and 200 nm in diameter and have a double-layered lipid membrane [11]. The biomolecules carried on exosomes have widely varying based on derived cells and isolate method. There are some ubiquitous proteins can distinguish exosomes from other extracellular vesicles, including tetraspanin proteins (CD81, CD9 and CD63), the exosome biogenesis related proteins (Hrs, flotillin, Tsg101), heat shock proteins (HSP70 and HSP90) and cytoskeletal proteins [12]. The genetic characterization and composition of exosomal nucleic acids are still not fully understood, but it is well known that exosomes can deliver genetic material such as miRNAs for therapeutic purposes [9]. The plasma membrane of exosomes is more robust and resistant to degradation than derived cells, probably due to the increased content of cholesterol, sphingolipids, and desaturated lipids [13]. Exosome formation is a sophisticated process. First, the cell membrane deforms and endocytoses to form intracellular endosomes. Then, the endosomes bud into intraluminal vesicles called multivesicular bodies (MVBs). Finally, MVBs fuse with the cell membrane, releasing exosomes into the extracellular [14,15]. Although there is no uniform standard, exosomes can be isolated using various techniques such as ultracentrifugation, immunoaffinity, microfluidic, precipitation technologies [12]. The most commonly isolate method is ultracentrifugation. Exosomes are isolated from cell debris, apoptotic bodies, and other large vesicles based on their different densities which requires ultra-high centrifugation speeds of up to 100,000 g [16]. The quantification methods of exosomes including nanoparticle tracking analysis (NTA), electron microscopy and dynamic light scattering. NTA provide direct quantification without relying on a specific maker but requires expensive instruments [17]. Electron microscopy is the most popular method for exosome characterization and quality assessments following isolation [18]. Exosomes stored in phosphate-buffered saline at −80 °C have improved stability and are further protected from cryodamage by the addition of trehalose and specific preservatives [19,20].

Exosomes derived from different cell types have been shown to play an important role in various stages of diabetic wound healing. However, due to limitations such as low production, the application prospect of exosomes may be restricted, and the advantages of exosomes-simulated vesicles are gradually being revealed. This review presents an overview of the significant and potential value of exosomes and membrane vesicles from diverse cell sources in diabetic foot wound healing.

## 2 Cell-based therapy and exosome-based therapy on regenerative medicine

Stem cells have been extensively researched in the field of regenerative medicine over the past few decades, demonstrating potential applications due to their regenerative properties [[21], [22], [23]]. However, there are many challenges associated with translating cellular therapy into the clinic [24]. Environmental dependence is one of the limitations for the clinical application of stem cells. When extracted and separated from their niche, the viability and quantity of stem cells may be negatively impacted [25]. Additionally, uncertainty arises regarding the clinical efficacy of stem cells due to the inability of preclinical experiments to fully replicate the physiological and genetic environments identical to human clinical conditions [26]. Another obstacle is the effect of long-term storage and freeze-thaw cycles on cell numbers and viability [27]. Therefore, the establishment of standard preservation procedures is necessary. There is a controversial issue concerning the potential tumorigenicity [[28], [29], [30]]. Stem cell transplantation may increase the risk of tumor growth and metastasis, which has been widely discussed in the field of breast reconstruction. Co-cultured adipose-derived stem cells and several human breast cancer cell lines revealed the ability of stem cells to enhance malignant features of cancer cells [30]. Ethical issues in stem cell therapy have always been controversial. While autologous stem cell transplantation mitigates the ethical risk, there are still difficulties in large-scale clinical translation [31,32].

Stem cell-based therapy was once considered a very promising emerging therapy for diabetic foot [33]. However, it also faces challenges as mentioned above. The most appropriate cell type, dosages and route of administration in clinical applications remain unclear [34]. Although mesenchymal stem cells (MSCs) can induce diabetic foot wound healing, ultrasound and dermoscopy have shown that the collagen density and epidermal thickness in areas of healed ulcers had not returned to normal, which could lead to a poor long-term prognosis [35]. To solve these problems, researchers have observed that secretome engineering, especially the use of exosomes, may enable cell-free therapy [11,33,36].

Compared to cell-based therapy, cell-derived exosomes have the ability to improve and regenerate a wide range of damaged tissues due to their stability and safer delivery of signaling molecules, such as bone, cartilage, cutaneous wounds and vascular tissue [37,38]. As nanoscale vesicles, exosomes can cross biological barriers, evade immune recognition, and target specific cells [39]. Exosomes from antigen-presenting cells demonstrate low toxicity and immunogenicity [40]. Exosomes loaded with specific small molecules, such as miRNA and drugs, can improve therapeutic efficiency due to their biocompatibility and stability [38]. Unique components on the surface of exosomes allow for direct fusion of the membrane with the target cell, facilitating efficient cellular targeting [[41], [42], [43]]. MSC-derived exosomes (MSC-Exos) offer significant advantages over MSCs themselves by effectively minimizing infusion-associated toxicity [44].

Numerous systematic reviews and studies have reported the advantage of exosome-based therapy for cutaneous wound healing [[45], [46], [47]]. Moreover, various studies have further pointed to the benefit of exosomes on diabetic wounds [10,[48], [49], [50], [51], [52], [53], [54]]. In conclusion, the comparison of cellular and exosome-based therapies demonstrates the superiority of exosome-based treatment in the context of treating and managing diabetic foot (Table 1).

**Table 1:** Comparison of cell-based and exosome-based therapies.

Cell-based therapy	Exosome-based therapy
Environmental dependence [25]	Biocompatibility and stability [36,38,39]
Uncertainty of clinical efficacy [26]	Efficient cellular targeting [[41], [42], [43]]
Storage and transportation difficulties [27]	Low immunogenicity [37,39,40]
The potential tumorigenicity [[28], [29], [30]]	Safety [39,44]
Ethical issues [31,32]	Capacity to cross biological barriers [37,39]
Infusion-associated toxicity [44]	Low toxicity [36,40,44]
	Loaded with specific small molecules [36,38]

## 3 Stem cells-derived exosomes

### 3.1 Mesenchymal stem cells-derived exosomes

**Fig. 1:**
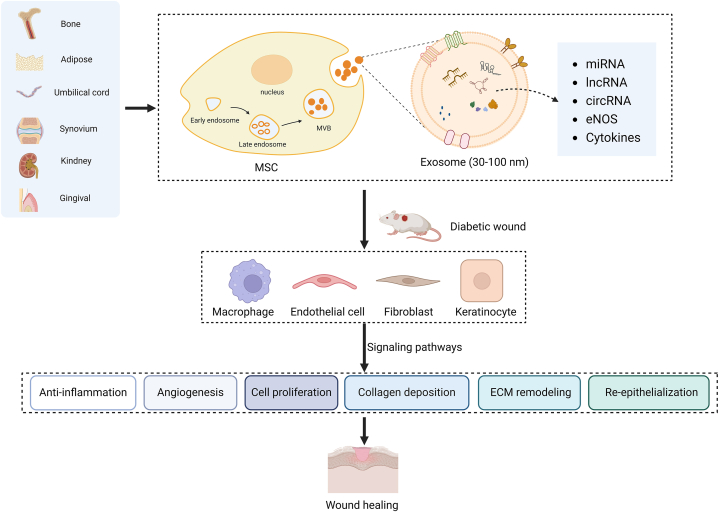
Therapeutic potential of mesenchymal stem cell-derived exosomes in promoting wound healing in diabetic models.

Mesenchymal stem cells possess the remarkable ability to self-renew and differentiate into various cell types [23,55]. Moreover, growth factors, cytokines, and collagen are also released, which facilitate wound healing and tissue regeneration [23]. This unique combination of properties makes mesenchymal stem cells an attractive candidate for therapeutic applications in the context of wound healing and tissue repair [24,46]. Consequently, based on the therapeutic potential, mesenchymal stem cells are considered as a promising therapy for enhancing wound healing in individuals with DFUs [56,57]. However, in the field of biomedicine, using stem cells presents ethical and potential safety issues [24]. Numerous studies indicated that mesenchymal stem cells promote wound healing through two different mechanisms [44,58]. First, mesenchymal stem cells directly differentiated into fibroblasts, myofibroblasts, and endothelial cells to promote angiogenesis and tissue repair [58]. Second, mesenchymal stem cells could release various bioactive molecules through paracrine effects to enhance cell proliferation and migration. In this context, mesenchymal stem cells-derived exosomes (MSCs-Exos) are the major delivery system [36,59]. MSCs-Exos, with a diameter ranging approximately 30–100 nm, maintain their integrity and biocompatibility through lipid bilayers, while the surface-modified proteins enhance their recognition and targeting capabilities. Moreover, abundant RNAs in MSCs-Exos can regulate receptor cells' transcription and translation [37]. Therefore, therapy based on MSCs-Exos provides an effective strategy for DFUs, mimicking the effects of stem cells while circumventing various limitations associated with stem cell therapy (Table 1). MSCs-Exos are involved in almost all stages of diabetic wound healing, including anti-inflammation, angiogenesis, collagen deposition, epithelial regeneration, and scar repair (Fig. 1).

#### 3.1.1 Bone marrow mesenchymal stem cells-derived exosomes

Bone marrow mesenchymal stem cells (BMSCs), the adult stem cells with low immunogenicity, are widely presented in the bone marrow, and capable of self-renewal and differentiation into diverse types of tissue cells. BMSCs secrete vascular endothelial growth factor (VEGF), epidermal growth factor (EGF), and interleukin-6 (IL-6) to improve the microenvironment and regulate inflammation, immunity, fibrosis, and apoptosis, thereby promoting the angiogenic response to accelerate tissue recovery [60]. BMSCs-derived exosomes inherit these functions, primarily promoting the proliferation of endothelial cells and keratinocytes [61]. They downregulate miR-383 to increase the expression of its target factor VEGFA, promoting endothelial cell proliferation, migration, and angiogenesis, while inhibiting endothelial apoptosis under hyperglycemic conditions, thus facilitating skin wound healing in diabetic mice [52]. They could also promote the proliferation and migration of fibroblasts by downregulating miR-152-3p, mediating phosphatase and tensin homolog (PTEN) to prevent fibroblast apoptosis and inflammation, thereby accelerating DFUs healing [62]. BMSCs-derived exosomes as drug delivery systems possesses excellent biocompatibility and target recognition capabilities to improve the healing of diabetic wounds. MiR-146a-5p can be loaded into BMSCs-derived exosomes by electroporation technology, which facilitates anti-inflammatory macrophage polarization by blocking tumor necrosis factor receptor-associated factor 6 (TRAF6) expression and promote endothelial cell proliferation, migration, angiogenesis and re-epithelialization in hyperglycemia [63]. Preconditioned MSCs with chemical or biological factors may enhance the biological activity of MSCs-derived exosomes. Exosomes derived from atorvastatin pretreated BMSCs can activate the AKT/eNOS pathway both *in vivo* and *in vitro* by upregulating miR-221-3p, increasing vascular endothelial growth factor levels and promoting angiogenesis, thus exhibiting excellent wound healing promotion capabilities in diabetic wound [64]. Melatonin pretreated hBMSC-derived exosomes (MT-Exos) upregulate PTEN expression and inhibit AKT phosphorylation, increasing M2 macrophage polarization and inhibiting the inflammatory response, ultimately improving diabetic wound healing [51]. By stimulating the PI3K/AKT/eNOS signaling system, exosomes derived from pioglitazone-treated MSCs increase endothelial cell functions and angiogenesis, thus promoting diabetic wound healing [65]. Previous research has indicated that hypoxic BMSCs could exacerbate hypoxia-inducible factor (HIF)-1α-mediated transforming growth factor (TGF)-β1 secretion, downregulate SMAD2 phosphorylation levels, induce keratinocyte proliferation and migration *via* keratinocyte autophagy, re-epithelialization to accelerate diabetic wound healing [66]. A recent study has shown that exosomes secreted by hypoxic BMSCs have similar effects. They target MAPK-activated protein kinase 2 (MAPKAPK2) through miR-4645-5p to inhibit AKT-mTORC1 signaling pathway in keratinocytes [67].

#### 3.1.2 Adipose-derived mesenchymal stem cell exosomes

Adipose-derived mesenchymal stem cells (ADSCs) are widely available and readily accessible multipotent adipose stem cells whose regenerative capacity has been demonstrated for cutaneous wound repair [24,[68], [69], [70]]. Exosomes from ADSCs also facilitate wound healing *via* a wide range of mechanisms, including anti-inflammation, anti-apoptosis, epithelial and vascular regeneration promotion, and reduction of scar formation [[71], [72], [73], [74], [75]]. Compared to ADSCs, exosomes from ADSCs are easier to store, more stable, and more biocompatible, which have been demonstrated in diabetic wound repair [45,76,77].

Studies indicated that ADSCs-Exos could inhibit cytotoxic T cells activation and proliferation, reduce IFN-γ production [78], and induce anti-inflammatory M2 macrophage polarization [79], making them a potential therapy for inflammation-related diseases. Additionally, hypoxia-induced ADSCs-Exos could upregulate miR-21-3p, miR-126-5p, miR-31-5p, and downregulate miR-99b and miR-146a to inhibit inflammation *via* the PI3K/AKT signaling pathway, serving as a strategy to promote diabetic wound healing [80]. They can also transfer circ-Snhg11 by the miR-144-3p/HIF-1α axis to promote M2 macrophage polarization and attenuates inflammatory factor secretion and cell apoptosis, eventually contributing to the diabetic wound healing [81]. Furthermore, ADSCs-Exos have been demonstrated to possess a remarkable protective effect on endothelial progenitor cells damaged by chronic hyperglycaemia, resulting in cell proliferation and angiogenesis [81,82], with the overexpression of nuclear factor-E2-related factor 2 (Nrf2) enhances its protective effect, as validated in diabetic rat wound [82]. ADSCs-Exos also reduce ROS production in human umbilical vein endothelial cells (HUVECs) and promote angiogenesis by modulating the mitochondrial deacetylase Sirtuin 3/superoxide dismutase (SIRT3/SOD2) pathway [82]. In diabetic mice, ADSCs-Exos stimulate monocyte/macrophage secretion of TGF-β1 and promote fibroblast function by activating the Smad3 signaling pathway, thereby accelerating diabetic wound healing [53]. Similarly, Interferon regulatory factor 1 (IRF1)-overexpressing rat ADSCs exosomes promote fibroblast proliferation, migration, and angiogenesis by regulating the miR-16-5p/SP5 axis [83]. Furthermore, exosomal vimentin from adipose progenitor cells have been shown to enhance fibroblast adaptation to external stress and inhibit stress-induced cell apoptosis [84]. Knockdown of low-density lipoprotein receptor-related protein 1 (LRP1) in fibroblasts and the use of an AKT pathway inhibitor can reverse the pro-healing effects of ADSCs-Exos on diabetic wounds, suggesting that HSP90 may be involved in the mechanism [49]. The high expression of miRNA-21 in ADSCs exosomes enhances migration and proliferation of HaCaT cells through the PI3K/AKT pathway, promoting extracellular matrix remodeling and significantly accelerating wound healing [85]. To enhance the abundance of miRNAs, miR-21-5p is loaded into hADSCs-Exos with electroporation technology to achieve more efficient therapy [86]. Exosomes can be loaded with other materials to accelerate wound healing synergistically. Hydrogel dressings have been extensively studied and utilized in wound repair due to their biocompatibility, biodegradability, and modifiability. The controlled release of exosomes from these dressings contributes to promoting diabetic wound healing [87]. Combining ADSCs-Exos with a multifunctional antimicrobial hydrogel to create FHE@exo has been shown to significantly promote endothelial cell function, increase skin adherence and reduce scarring in diabetic wound healing, proving to be more effective than single exosome therapy [88]. Incorporating exosomes from ADSCs into extracellular matrix hydrogel for topical treatment increases exosome concentration in wounds and accelerates diabetic wound healing by inhibiting inflammation, and promoting angiogenesis and cell proliferation and migration [89].

#### 3.1.3 Umbilical cord mesenchymal stem cells-derived exosomes

Various cytokines and growth factors can be synthesized and secreted by Human umbilical cord mesenchymal stem cells (Huc MSCs), stimulating other cells to proliferate. Cytokines IL-6 and IL-8 contained in Huc MSCs exosomes can promote cell proliferation and prevent oxidative stress-induced apoptosis by activating ERK1/2 and p38 [90]. In addition, Huc MSCs exosomes transport abundant miRNAs, including miR-181, miR-21, and miR-146a, which can inhibit inflammatory responses at the wound site [91]. Moreover, endothelial cells activated by Wnt4 molecules accelerate proliferation and migration, resulting in rapid neovascularization [92]. In diabetic wound healing, Huc MSCs exosomes can also improve endothelial cell oxidative stress damage and promote angiogenesis [93]. Another study demonstrated that exosomes isolated from Huc MSCs promoted the proliferation of HUVECs and mouse embryonic fibroblasts *in vitro*, and *in vivo*, such exosomes could promote diabetic wound repair by inducing anti-inflammatory macrophages, promoting collagen deposition, and angiogenesis [94]. Meanwhile, the biological activity of derived exosomes can be enhanced by pretreating MSCs with genetic engineering techniques or biochemical factors. For example, genetically engineered Huc MSCs exosomes loaded with a large amount of eNOS under blue light irradiation significantly improved the biological function of cells grown in high levels of glucose, reduced oxidative stress-induced inflammatory factor expression and apoptosis, regulated the associated immune microenvironment and enhanced matrix remodeling, thereby promoting angiogenesis and tissue repair in chronic diabetic wounds [95]. In contrast, Huc MSCs exosomes pretreated with Nocardia rubra cell wall skeleton (Nr-CWS) stimulated the expression of circIARS1, which mediated the miR-4782-5p/VEGFA axis to promote endothelial cell proliferation and migration, thereby offering a potential strategy for diabetic wound treatment through tissue angiogenesis [96]. In a diabetic rat model, the topical application of Huc MSCs-derived exosomes wrapped in thermosensitive PF-127 hydrogel to full-thickness skin wounds resulted in increased Ki67 and CD31 expression, improved granulation tissue regeneration and upregulated VEGF and TGFβ-1 expression, thus promoted diabetic wound healing [97]. Furthermore, combining Huc MSCs-derived exosomes with polyvinyl alcohol (PVA)/alginate (Alg) nanohydrogel increased the expression of smooth muscle actin (SMA), the scavenger receptor, class B type 1 (SR-B1), and CD31, upregulated VEGF levels through ERK1/2, promoted HUVECs and angiogenesis, eventually facilitating the wound healing in diabetic rats [50]. A recent study has indicated that exosomes prepared from fresh human Huc MSCs, which combined with chitosan nanoparticles, bioactive glass (BG), and titanium dioxide (TiO2) in a composite hydrogel, can significantly enhance angiogenesis *via* VEGFA and VEGFR2, and accelerate full-thickness skin defect repair in diabetic mice with anti-inflammatory and antimicrobial activities [98].

#### 3.1.4 Other mesenchymal stem cells-derived exosomes

Studies have demonstrated that synovium mesenchymal stem cells (SMSCs) could significantly enhance the proliferation of fibroblasts. SMSCs-derived exosomes overexpressing miR-126-3p can stimulate the proliferation of human fibroblasts and microvascular endothelial cells. In the diabetic rat model, these exosomes suggest accelerated epithelial regeneration of wounds by promoting cell proliferation and angiogenesis [99]. Exosomes derived from human urine-derived stem cells can promote endothelial cell function by transporting pro-angiogenic protein DMBT1, which may be one of the most promising strategies for accelerating the healing of diabetic wound [100]. Gingival MSCs-derived exosomes have been found to regulate the Wnt/β-catenin signaling pathway, promoting proliferation, migration, and angiogenesis of HUVECs in a hyperglycemic environment, while nanomaterial-encapsulated exosomes show more pronounced pro-healing effect on full-thickness wounds in diabetic mouse model, with potential for clinical translation [101]. Human amnion MSCs exosomes downregulate LATS2 expression *via* miR-135a and promote fibroblast migration, thereby accelerating skin wound healing in rats [102].

### 3.2 Other stem cells-derived exosomes

#### 3.2.1 Endothelial progenitor cells-derived exosomes

The endothelial progenitor cells (EPCs) are multipotent stem cells that can differentiate into the endothelial cells of the body. Circulating EPCs express CD34, VEGFR2, and CD133 [103], and possess unique angiogenic and reparative functions that promote dermal wound healing [104,105]. The cellular decline and dysfunction of EPCs in diabetes lead to delayed wound healing. Targeting EPCs could accelerate the healing [106]. Exosomes derived from EPCs possess the same functions as the original cells and hold promising potential for direct application in regenerative medicine [107,108]. It has been shown that EPCs-Exos can activate the Erk1/2 pathway in vascular endothelial cells, promoting cell proliferation, migration, and vascular formation, and thus facilitating diabetic skin wound healing in rats [109]. Further study has indicated that EPC-Exos may enhance endothelial cell function *via* highly specific miRNA expression and downstream gene regulation [110]. A recent study has demonstrated that EPCs-Exos may inhibit ferroptosis in HUVECs and endothelial damage by upregulating miR-30e-5p, suppressing specificity protein 1 (SP1), and activating the adenosine monophosphate-activated protein kinase (AMPK) pathway [111]. In another study, EPCs-derived exosomes with miR-126-3p overexpression not only rescued the impaired proliferation and migration capabilities of HUVECs caused by hyperglycaemia but also inhibited endothelial cells pyroptosis by targeting the PI3KR2/SPRED1 signaling pathway [112]. It was found that EPC-Exos regulate the function of vascular endothelial cells and inhibit the expression of PPARG by increasing the level of miR-182-5p, thus promoting the proliferation and migration of HaCaTs and inhibiting apoptosis in a high glucose environment. These effects have been validated in diabetic mice [113].

#### 3.2.2 Epidermal stem cell (ESCs) exosomes

Epidermal stem cells (ESCs) show extensive potential in wound regeneration, due to the abundance, ease of access, and minimal ethical concerns [[114], [115], [116]]. Research on diabetic wound healing indicated that ESCs expedite healing by promoting inflammation resolution, M2 macrophage polarization, vascularization, as well as cell proliferation and migration [54]. ESCs-Exos act as intercellular communication mediators, exhibiting similar functions with higher biocompatibility, lower immunogenicity, and simpler storage and transportation [54]. Mechanistically, ESCs-Exos primarily exert their effects by transferring of miRNA. For instance, ESCs-Exos enriched with miR-142-3p and miR-425-5p inhibit myofibroblast differentiation, and reduce scarring by decreasing TGF-β1 expression in dermal fibroblasts during wound healing [117]. Furthermore, the PI3K/AKT and MAPK signaling pathways play a role in ESCs-Exos' regulation of diabetic wound healing [54]. Chronic hyperglycemia causes endothelial cell dysfunction and excessive autophagy-induced apoptosis. ESCs-Exos riched with miR-200b-3p target the synapse defective rho GTPase homolog 1/RAS/ERK pathway to alleviate endothelial cell autophagy and apoptosis, with validation of their angiogenesis in db/db mouse dorsal wounds [118]. Another study confirmed that ESCs-Exos riched with miR-203a-3p could downregulate the negative regulator SOCS3 and activate the JAK2/STAT3 signaling pathway to promote M2 macrophage polarization [119]. To enhance the use of exosomes, ESCs-Exos can be merged with other materials. For example, exosomes loaded with VH298, an E3 ubiquitin ligase inhibitor, could enhance endothelial cells function and vascularization in wounds by activating the HIF-1α/VEGFA signaling pathway. Encapsulating exosomes in gelatin methacryloyl (GelMA) hydrogel for sustained release reinforces this effect, offering a new functional dressing for the treatment of diabetic wounds [120].

#### 3.2.3 Human amniotic epithelial cells (hAEC) exosomes

Amniotic epithelial cells are low-immunogenic multipotent stem cells that produce growth factors, vascular regulatory factors, and anti-inflammatory factors that aid in skin wound healing. Isolating exosomes from amniotic cells has facilitated their use in regenerative medicine [121]. HAECs promote M2 macrophage polarization and endothelial cell function, and topical injection into full-thickness cutaneous wounds of db/db mice has confirmed the role in promoting diabetic wound healing [122]. Studies on hAEC-Exos reveals that they accelerate wound healing by stimulating fibroblast migration and proliferation through the delivery of exosomal miRNAs [123]. Another study showed that hAECs-Exos, activate the PI3K-AKT-mTOR pathway *via* miRNA, significantly promoting the proliferation and migration of human fibroblasts (HFBs) and the vascular regeneration activity of HUVECs, thereby inducing diabetic wound healing [124]. Additionally, hypoxia-induced hAECs-Exos are readily absorbed by keratinocytes and fibroblasts, enhancing cell proliferation and epithelial regeneration, reducing scar formation in normal wounds, and aiding in angiogenesis [125]. However, further investigation is needed to ascertain if they exert a similar impact on diabetic wounds.

## 4 Hemocyte-derived exosomes

### 4.1 Platelet-derived exosomes

Numerous studies have demonstrated that platelets can release various growth factors to regulate inflammation, stimulate angiogenesis, and promote granulation tissue growth during the wound healing process, starting from the hemostatic phase [126]. In 2023, the updated IWGDF recommended the adjunct use of platelet patches to promote the healing of diabetic foot ulcers [6]. Platelets and platelet-derived products play a significant role in diabetic wound healing, we have successfully utilized platelet-rich plasma (PRP) to accelerate diabetic ulcers healing in many cases [127]. Furthermore, we have demonstrated the antimicrobial, anti-inflammatory, and pro-cellular proliferative effects of PRP in an *in vitro* model of diabetic infected wound [128]. Subsequently, we isolated nanoscale cup-shaped vesicles, known as platelet-rich plasma exosomes (PRP-Exos), under different preparation conditions, which carry various proteins, mRNA, microRNA, and other bioactive substances from PRP. *In vitro* experiments demonstrated that PRP-Exos activated by calcium gluconate/thrombin mixture significantly promoted the proliferation and migration of HUVECs and induced tube formation through the AKT/ERK signaling pathway [129]. Therefore, PRP-Exos have the potential to promote healing of wounds. Furthermore, our study has revealed that sphingosine-1-phosphate enriched in platelet-rich plasma exosomes (PRP-Exos-S1P) transmits signals by binding to sphingosine-1-phosphate receptor 1 (S1PR1) on the membrane of skin vascular endothelial cells, thereby activating the protein kinase B/fibronectin 1 (AKT/FN1) signaling pathway to promote angiogenesis and repair in diabetic wounds [130]. Our recent study confirmed that PRP-Exos improve the healing of diabetic wound by transporting miR-26b-5p, which targets MMP-8 and subsequently inhibits NETs [131].Several recent studies have supported our findings. Guo et al. showed that PRP-Exos contain growth factors such as bFGF, VEGF, PDGF-BB and TFG-β, which promote the proliferation and migration of fibroblasts and HMEC-1 cells through YAP activation. Moreover, the pro-angiogenic and re-epithelialization effects of PRP-Exos were verified in diabetic rats [132]. Chen et al. found that lncRNA MALAT1 is downregulated in fibroblasts of diabetic foot ulcers, and the overexpression of MALAT1 by PRP-Exos enhances fibroblast viability, inhibits apoptosis and pyroptosis. This suggests the MALAT1-mediated signaling pathway probably contribute to the promoting effect of PRP-Exos on diabetic foot ulcer healing, potentially serving as a new target for diabetic foot treatment [133]. However, further exploration of its downstream regulatory mechanisms is necessary. Similarly, using PRP-Exos, Yang et al. demonstrated *in vitro* and *vivo* therapeutic potential for diabetic wounds. They hypothesized that PRP-Exos might prevent high glucose-induced ROS-dependent cell apoptosis through the PDGF-BB/JAK2/STAT3/Bcl-2 signal axis to promote fibroblast function and accelerate diabetic wound healing [134].

Additionally, novel gel dressings obtained by combining PRP-Exos, drugs, and materials such as hydrogels through bioengineering can alleviate the limitations of single drug administration and achieve complementary advantages, holding promising prospects for application in diabetic wounds. Xu et al. embedded curcuma zedoaria polysaccharide (ZWP) and PRP-Exos in chitosan/silk hydrogel sponge and verified its potential for diabetic wound healing in rats was superior to single drug treatment [135]. Zhu et al. loaded platelet-derived extracellular vesicles (PDEVs) and resveratrol (RES) nanoparticles in gelatin methacrylate/silk fibroin methacrylate (GelMA/SFMA) composite hydrogel, which suppressed iNOS expression in macrophages and promoted tube formation in HUVECs *in vitro*. In the diabetic mouse wound model, this gel dressing reduced the expression of iNOS and TNF-α, and upregulated anti-inflammatory factors such as Arg-1 and TGF-β1, thereby accelerating wound recovery [136]. They speculated that this regulatory mechanism may be related to the extracellular purinergic signaling pathway but did not further elucidate it. Bakadia et al. developed silk protein (SP)-based novel generation double-crosslinked hydrogel including PRP or PRP-Exos, with excellent mechanical properties, which enabled the sustained release of growth factors (GFs) and exosomes to accelerate the healing of diabetic wounds through the upregulation of GFs, the downregulation of MMP-9, and the promotion of *anti*-NETotic, angiogenesis, and re-epithelialization [137]. Hao et al. prepared polymer-coordinated hydrogels containing platelet-derived extracellular vesicles (pEV) and reduced graphene oxide (rGO), which exhibited excellent mechanical stability and biocompatibility *in vitro* and showed strong macrophage polarization and ROS scavenging. These properties were validated in diabetic rats, where GelAlg@rGO-pEV regulated the immune responses and inflammation, and promoted angiogenesis and hair follicle regeneration, thus accelerating the healing of chronic wounds [138]. Likewise, Shu et al. designed fiber-reinforced gelatin (GEL)/β-cyclodextrin (β-CD) therapeutic hydrogels encapsulating PRP-EXOs, which offer a biocompatible microenvironment and active ingredients for cell adhesion, proliferation, and skin tissue regeneration. This provides a new therapeutic platform for diabetic wound healing by modulating autophagy and inhibiting apoptosis of HUVECs and human skin fibroblasts (HSFs), inducing vascularization, collagen deposition, and re-epithelialization [139].

Like stem cells exosomes, the isolation and purification processes of platelet-derived exosomes are complex and time-consuming, and there is no efficient large-scale extraction method. Additionally, most previous studies were limited *in vitro* and murine animal models, so the application of platelet exosomes in clinical therapy is urgently needed to explore. A recent study provided the first evidence of the safety and therapeutic utility of clinical-grade pEVs in humans. They extracted clinical-grade pEVs on a large-scale using Ligand-based Exosome Affinity Purification chromatography. The safety of pEVs were confirmed in a randomized placebo-controlled Phase I clinical trial in healthy volunteers (ACTRN12620000944932) [140]. The trial involved a single subcutaneous injection. While the outcome indicated no adverse effects, there is no guarantee that repeated injections and systemic administration will produce the same result. The result also indicated no difference in the recovery times between the experimental and placebo groups, which may be related to the small sample size and the participants' healthy physiology. In other word, further clinical trials are needed to confirm the efficacy and safety of different route of administration and repeated doses. At the same time, the therapeutic effect of pEVs on delayed healing wounds such as DFUs should be verified. Similarly, an ongoing clinical trial (NCT06429033) aims to evaluate the safety and histological profile of purified exosome product (PEP) in healthy adults *via* hypodermic injection. The safety and efficacy of topical PEP in DFUs is being validated in an ongoing Phase 2a clinical trial (NCT06319287).

### 4.2 Leukocyte-derived exosomes

The persistent chronic inflammation in the diabetic wound microenvironment, where leukocyte dysfunction plays a critical role, contributes to delayed wound healing [[3], [4], [5]]. According to the IWGDF 2023 guidelines on diabetic foot ulcers, topical supplementation of autologous leukocytes can accelerate healing [6]. Immune cell exosomes, derived from sources such as macrophages and neutrophils, have been reported as potential factors in this process. Continuing to explore the potential of leukocyte-associated exosomes in diabetic wound healing is an important area of research.

#### 4.2.1 Macrophage-derived exosomes

Macrophages are critical in wound healing by regulating inflammation, angiogenesis, and fibrosis [141]. However, in diabetic wounds, macrophage dysfunction, impaired polarization of M2 anti-inflammatory macrophages, increased pro-inflammatory factors and decreased anti-inflammatory factors lead to challenges during the healing process [3]. Therefore, improving macrophage function, promoting the polarization of M1-to-M2 macrophages, and activating the transdifferentiation of macrophages to fibroblasts can accelerate chronic diabetic wound healing [142]. Theoretically, exosomes derived from M2 macrophages (M2-Exos) have similar anti-inflammatory and pro-angiogenic functions as M2 macrophages. Studies have shown that M2-Exos can accelerate wound healing in diabetic wounds by promoting the switch of M1 to M2 macrophages, enhancing angiogenesis, re-epithelialization, and collagen deposition [143]. Chronic diabetic wounds with an imbalance between proinflammatory and anti-inflammatory responses could benefit from this treatment strategy. Studies on diabetic fracture healing have shown that M2-Exos induce the transformation of M1 macrophages into M2 macrophages through the simulation of the PI3K/AKT pathway [144]. It was shown that encapsulation of M2-Exos in a biodegradable polyethylene glycol (PEG) hydrogel resulted in sustained release of exosomes, maximizing their therapeutic effects in skin wound healing [145]. A specific source of macrophage-derived exosomes secreted from lean mouse adipose tissue can modulate db/db murine macrophage polarization *via* miR-222-3p, promoting rapid healing of diabetic wounds [146].

In the diabetic rat model, macrophage-derived exosomes exert anti-inflammatory effects by inhibiting the secretion of pro-inflammatory enzymes and cytokines and accelerate wound healing by inducing endothelial cell proliferation and migration, improving vascularization and re-epithelialization [147]. Comparison of M0-Exos, M1-Exos, and M2-Exos revealed that M2-Exos promote HUVEC migration and tube formation, while M1-Exos exhibit the opposite effect. High-throughput sequencing showed that miRNA-155-5p is highly expressed in M1-Exos, inhibiting the expression of growth differentiation factor 6 protein, thereby blocking angiogenesis of HUVECs [148]. Therefore, M2-Exos and miRNA-155-5p inhibitors may be effective strategies for the treatment of diabetic foot ulcers. However, the therapy with exosomes face limitations such as short half-life and instability. To overcomes these challenges, researchers have designed various multifunctional hydrogels to accelerate diabetic wound repair by promoting angiogenesis and re-epithelialization by sustained release of M2-Exos and growth factors [149,150]. An integrated hydrogel system study showed that combining anti-swelling hydrogel with anti-inflammatory M2-Exos and gold nanorods (AuNRs) photothermal effects effectively inhibits bacteria and inhibit inflammation, reduces reactive oxygen species, and angiogenesis for efficient synergistic diabetic wound treatment [151]. Another wound dressing system based on double-layer microneedles (MEs@PMN), encapsulating M2-Exos and polydopamine nanoparticles, enhances M2 macrophage polarization and can be combined with mild photothermal therapy to suppress inflammation and improve vascular regeneration, thereby accelerating diabetic wound healing [152]. Curcumin-loaded macrophage exosomes (Exos-cur) as a novel pro-healing biomaterial with better stability, anti-inflammatory, and antioxidant bioactivities were experimentally shown to inhibit inflammation, upregulate the expression of wound healing-related molecules, promote HUVEC proliferation, migration, and vascularization, optimize re-epithelialization, inhibit oxidative stress, thus accelerate diabetic rat wound healing *via* the Nrf2/ARE pathway [47]. A recent study reported that two-dimensional carbide (MXene)-M2-Exo nanohybrids (FM-Exo) can continuously release exosomes for up to 7 days, exhibiting a broad-spectrum antibacterial activity and immunosuppression. Mechanistically, FM-Exo can promote the function of fibroblasts and endothelial cells by activating the PI3K/AKT signaling pathway and significantly induce macrophage polarization [153].

#### 4.2.2 Other leukocyte-derived exosomes

Neutrophils are the most abundant circulating leukocyte cells in the human body and served as the primary immune cells against pathogens with intrinsic phagocytosis [154,155]. Neutrophil-derived exosomes have been proven to be divided into anti-inflammatory and pro-inflammatory types [156]. Anti-inflammatory neutrophil-derived microvesicles (NDMVs) contain miR-126, miR-150, and miR-451a, which are taken up by monocytes to simulate M2 macrophage polarization, and may be considered as a therapeutic potential for chronic inflammatory diseases such as DFUs [157]. Dendritic cells (DCs), as the major antigen-presenting cells of the immune system, play a crucial role in initiating and regulating protective pro-inflammatory and immune responses [158]. DCs exosomes can promote angiogenesis and tissue regeneration by carrying and delivering a variety of miRNAs that modulate the function of other cells. For example, exosomes derived from mature dendritic cells (mDC-Exos) inhibit the Hippo signaling pathway by transferring miR-335 and target large tongue suppressor kinase 1 (LATS1), promoting the proliferation and osteogenic differentiation of BM-MSCs in rats with femoral defects [159]. Exosomes isolated from mouse bone marrow-derived DCs (DEX) significantly upregulate the expression of VEGF in cardiac microvascular endothelial cells (CMECs), to enhance CMEC tube formation. *In vivo*, DEX facilitated angiogenesis in mice by delivering miR-494-3p [160]. Therefore, DEX may represent a potential therapeutic strategy for diabetic wound healing. Additionally, exosomes derived from dendritic epidermal T cells (DETC) have been shown to accelerate re-epithelialization of skin wounds by promoting the proliferation of ESCs [161]. However, diabetic wounds often exhibit excessive inflammation and immune response. Further research and discussion are needed to effectively utilize these immune cell-derived exosomes to promote diabetic wound healing.

## 5 Endothelial cells-derived exosomes

Endothelial cells play a very important role in maintaining normal tissue physiology, as well as in acute and chronic inflammatory responses [162]. Targeting endothelial cells in diabetic wounds with persistent inflammation is an effective therapeutic strategy [163]. Therapy based on endothelial cell-derived exosomes may be another effective treatment modality to promote diabetic wound healing [164,165]. For example, hydrogel microneedle patches containing HUVECs-Exos achieve controlled release and transdermal delivery of exosomes around the wound, showing promise as a clinical treatment method for diabetic foot ulcers [164]. Another multifunctional acellular dermal matrix (ADM) hydrogel containing HUVEC-Exos has been proven to have the ability to promote diabetic wound healing [165]. In terms of mechanisms, hypoxic HUVECs-Exos can significantly upregulate lncHAR1B, which interacts with the transcription factor BHLHE23 to promote the expression of the KLF transcription factor 4, thereby ameliorate endothelial cell dysfunction and promote M2 macrophage polarization in diabetic wounds [48]. Overall, the specific role and molecular mechanism of HUVECs-exos in promoting diabetic wound healing are less studied and need further research.

## 6 Membrane vesicles

Membrane vesicles refer to artificial exosomes mimetic vesicles (EMVs) or extracellular vesicle mimetics [166,167], including cell membrane-coated synthetic nanovesicles, which serve as emerging biomaterials and drug delivery systems widely used in oncology, cardiovascular, immunology, and inflammatory disease research [[168], [169], [170], [171], [172]]. Compared with exosomes, membrane vesicles have a simpler preparation process, larger yield, and greater modifiability, which may lead to more efficient drug delivery efficiency [173]. Membrane vesicles are formed by extrusion, sonication, homogenization, and nitrogen cavitation [[174], [175], [176], [177]]. The most common method involves using an extruder to pressurize purified cell membranes or whole cells through polycarbonate membranes to prepare uniform-sized vesicles [178,179]. Similar to exosomes, various cells could be regarded as sources for drug-carrying membrane vesicles. Notably, nanoscale membrane vesicles derived from plants also have the potential as drug delivery systems due to their unique bioregulatory activity and editable nature [180]. In addition to obtaining membrane vesicles of single-cell origin, hybrid biomimetic membrane nanoparticles can be prepared using membranes from different cells [181,182].

EMVs are comparable to exosomes but have a hundred-fold higher yield and significantly higher levels of RNAs and proteins expression [183,184]. Another advantage of membrane vesicles is the varied modifiability, altering the expression of specific proteins or changing the ratio of cell membrane components by modulating the genetic information of the source cells or directly modifying the cell membrane to form specific membrane vesicles. Glycosylated platelet membrane vesicles prepared by co-incubating platelets with fucose and enzymes at room temperature have enhanced cell adhesion capabilities due to changes in membrane glycoprotein function [185]. The alteration of the cholesterol ratio can change membrane fluidity and rigidity. During vesicle preparation, adding free cholesterol directly to platelet membrane suspension and incubating it at room temperature for 30 min before extrusion results in more stable and drug-loaded platelet membrane vesicles enriched in cholesterol [176].

**Fig. 2:**
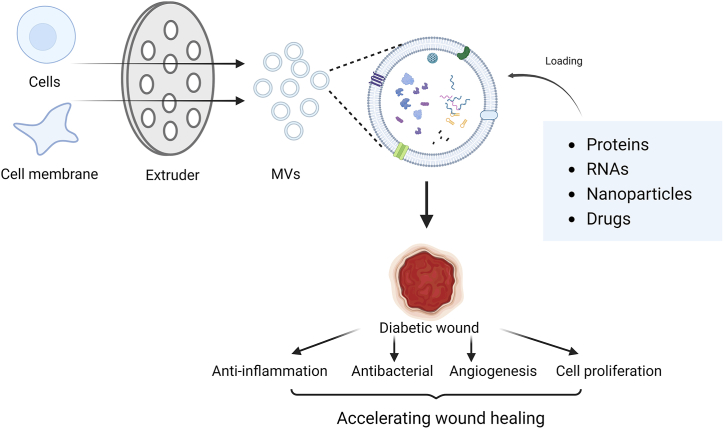
Continuous extrusion for cells or cell membranes with porous polycarbonate membranes could be utilized to produce vesicles with uniform size. These membrane vesicles load effective molecules to accelerate diabetic wound healing.

In the field of wound healing, membrane vesicles play a pro-healing role by transporting RNAs and proteins from source cells [184,186], as well as external drugs through co-extrusion [178,187], to regulate inflammation, angiogenesis, and re-epithelialization (Fig. 2). However, compared to exosomes, the research of membrane vesicles on wound healing, especially in diabetic wounds is limited, and further studies are urgently needed.

### 6.1 Stem cell membrane vesicles

The high expression of chemokine receptors on the membrane of stem cells endows them with the powerful anti-inflammatory and anti-injurious capabilities. Through bioengineering techniques, stem cells can be engineered to overexpress CXCR4, enhancing their homing effect on damaged tissues [188]. Stem cell membrane vesicles are considered to be a drug delivery system with significant advantages and clinical potential compared to others for promoting skin regeneration and wound healing [189]. Among them, mesenchymal stem cell membrane vesicles exhibit targeting and immune evasion properties, offering broad application potential in tissue regeneration, anti-tumor therapy, and anti-inflammatory research [190]. Local application of hUMSC EMV significantly induced regeneration of injured skin in mice, and it has been confirmed that EMV promoted the proliferation, migration, and tube formation of HUVECs *in vitro* experiments [184]. HUMSCs membrane vesicles conjugated with hydrogel scaffolds and were found to promote normal mouse wound healing more effectively than exosome hydrogels and proteomic analysis showed enrichment of mitochondrial-derived oxidative phosphorylation-related proteins [186]. Drug-loaded exosome mimetic vesicles were prepared by co-extrusion of the drug with induced pluripotent stem cell-derived endothelial cells (iPS-ECs) with abundant CXCR4, enabling homing to endothelial cells. These drug-loaded vesicles can promote endothelial cell function through the HIF-1α/VEGFA pathway to induce angiogenesis, and promote diabetic wound healing [178].

### 6.2 Platelet membrane vesicles

Platelet membrane vesicles have the ability to evade phagocytosis in the circulation due to cell membrane surface factors like CD47 [191]. Membrane vesicles engineered with glycosylated platelets and encapsulating interleukin-10 nanoparticles have the capacity to promote M2-type macrophage polarization to alleviate inflammation while promoting endothelial cell repair [185]. Platelet membrane encapsulated nanoparticles with bFGF and VEGFA gene plasmids can effectively target burn sites in rats *via* tail vein injection, and promote tissue healing [187]. Platelet membrane-coated therapeutic nanoparticles form multifunctional antibacterial agents (CSO@PM) with potent bactericidal and anti-inflammatory effects, as well as promote re-epithelialization and collagen deposition in wounds infected with drug-resistant bacteria, potentially aiding in the healing of diabetic foot ulcers [192]. In a recent study, Fe/Zn metal-organic frameworks were encapsulated in platelet membranes to create nanozymes with accelerated peroxidase-like activity. These nanozymes demonstrated robust antibacterial capabilities, notably suppressed levels of inflammatory cytokines, and facilitated angiogenesis. They accelerated skin wound healing when applied locally, as evidenced in a mouse model with infected full-thickness skin wounds [193]. However, further research is needed to determine their impact on diabetic wounds.

### 6.3 Leukocyte membrane vesicles

Neutrophil-derived membrane vesicles have a high affinity for the site of inflammation and have been used for drug delivery research in cancer and inflammatory diseases [194,195]. Membrane vesicles derived from HL-60 neutrophils form a drug delivery system that specifically targets inflamed brain endothelium in a mouse model of ischemic stroke and promotes the resolution of inflammation. This process is regulated by the expression of integrin β2 and adhesion molecule PSGL-1, both of which are expressed on neutrophil membranes [196]. Therefore, researchers believe that personalized nanoparticles formulated from neutrophil membranes have the potential to treat various inflammation-related diseases. Furthermore, human neutrophil membrane-derived nanovesicles loaded with resolvin D1 and ceftazidime specifically target inflammatory tissues and enhance the antibacterial effects, in a mouse model of bacterium-induced peritonitis [197]. In terms of systemic metabolism, studies have shown that drug-loaded membrane vesicles prepared by ultrasonication and co-extrusion of macrophage membranes with bovine serum albumin nanoparticles can effectively neutralize and remove inflammatory cytokines, as well as block immune and inflammatory cascade responses, to protect pancreatic islet cells and reduce insulin resistance, showing enormous potential in preventing and treating type 2 diabetes mellitus [198]. The use of neutrophil-derived exosome mimetics (PMNExo) for VEGF therapy delivery, combined with ECM hydrogels, has led to the development of injectable hybrid hydrogels with antimicrobial activity and wound healing properties, providing a prospective treatment platform for diabetic wound management [199]. Furthermore, the fusion of iPS-ECs membrane with M1 macrophage membrane to construct nanovesicles loaded with 4-octyl itaconate (4OI) can target M1 macrophages and endothelial cells, thereby promoting macrophage polarization and protecting endothelial cells, which in combination with injectable hydrogels, provides a novel strategy for promote diabetic wound repair [179].

## 7 Discussion and perspective

In conclusion, Exosomes have powerful pro-healing effects in diabetic wound and have safer and lower-immunity compared with cellular therapies. The toxicological characteristics of exosomes derived from human ADSCs have been evaluated through animal and cell experiments, demonstrating their safety for local treatment [200]. Furthermore, modification with genetic engineering techniques or chemical biofactors can increase the biological functionality of exosomes, thereby enhancing their therapeutical effect. Combining exosomes with hydrogels and nanomaterials can also accelerate wound healing, providing better therapeutic options for diabetic patients. In addition to regulating immune inflammation, angiogenesis, and cell proliferation, exosomes can also promote physiological processes of peripheral nerve, muscles and bone repair in the lower limbs. For example, ADSCs-Exos inhibit alkaline ceramidase 2 through miRNA-125b-5p to promote the proliferation and migration of myoblasts in diabetic mice, playing a crucial role in the repair of ischemic muscles in diabetic lower limbs [201].

Therefore, Exos have enormous potential as a therapeutic strategy to promote the healing of diabetic foot. However, despite the progress in preclinical experimental studies, there are still many challenges to be addressed before exosomes can be used in clinical settings. First, most of the current animal experiments are conducted based on acute diabetic wound models in mice and rats. The efficacy and safety of exosomes from different cellular sources need to be validated in animal models that closely resemble human anatomy to facilitate their use in future clinical trials. The safety and efficacy of exosomes in non-diabetic wound healing have been demonstrated in several completed clinical studies [140,202,203]. For example, a pilot case-control interventional study demonstrated the clinical efficacy of autologous serum-derived EVs in facilitating the healing process of chronic venous ulcers (CS2/1095/0090491) [203]. Additionally, ADSC-Exos showed both efficacy and safety in the treatment of acne scars in a double-blind randomized clinical trial [202]. However, their role in diabetic wound has not been validated in humans. Furthermore, exosomes from various cell types exhibit functional and property differences. The senescence phenotype SMCs-Exos may induce inflammaging as a side effect through their secretome [204]. The safety and efficacy of different administration routes may vary, requiring more short-term and long-term health safety assessments to confirm their safety. Additionally, issues regarding the large-scale production of exosomes and efficiency have yet to be determined. Currently, there is a lack of official worldwide standards for preparation, storage, and transportation of exosomes. The US Food and Drug Administration (FDA) has issued cautions that no exosome products are currently approved for routine clinical [205]. The regulation of exosomes is currently undergoing global improvement, and they should be regulated similarly to biological agents. Before being granted clinical approval, two key aspects need to be addressed. Firstly, there is need to understand the composition and efficacy of each type of exosomes. Secondly, legislation surrounding the regulation of exosomes needs to be refined.

Membrane vesicles have been becoming a novel delivery system for drugs and biomolecules due to their simple production process, abundant yield, high drug loading rate, and adjustability in regenerative medicine. How to utilize membrane vesicles to promote diabetic foot skin wound healing is still worth exploring and researching.

## CRediT authorship contribution statement

**Zhou Yang:** Conceptualization, Investigation, Writing – original draft. **Mengling Yang:** Investigation, Conceptualization, Writing – original draft. **Shunli Rui:** Funding acquisition, Investigation. **Wei Hao:** Visualization, Writing – review & editing. **Xiaohua Wu:** Validation, Writing – review & editing. **Lian Guo:** Project administration, Visualization. **David G. Armstrong:** Writing – review & editing, Conceptualization, Data curation. **Cheng Yang:** Funding acquisition, Investigation, Project administration, Writing – review & editing. **Wuquan Deng:** Supervision, Funding acquisition, Project administration, Resources, Writing – review & editing.

## Ethics statement

This narrative review did not involve any direct experiments on creatures, only summarized the existing data that has been published, and does not require the approval and review by the Ethics Commission. This review was completed in accordance with the ethical standard of *Heliyon*.

## Declaration of competing interest

We hereby declare that there are no conflicts of interest to report. This encompasses financial, consultative, institutional, and other forms of conflicts that might bias the work submitted. We understand the importance of this declaration in maintaining the integrity of the scientific record and assert that no such conflicts exist.

These disclosures do not alter our adherence to Elsevier policies on sharing data and materials in the review and publication of our manuscript. We have disclosed all information regarding any potential conflicts of interest to the best of our knowledge and believe.

Thank you for your consideration of our work. We trust that this declaration will assist the editorial process and contribute to the integrity of the scholarly record.
